# Measurement Invariance of Psychotic-Like Symptoms as Measured With the Prodromal Questionnaire, Brief Version (PQ-B) in Adolescent and Adult Population Samples

**DOI:** 10.3389/fpsyt.2020.593355

**Published:** 2021-01-21

**Authors:** Ulla Lång, Vijay Anand Mittal, Jason Schiffman, Sebastian Therman

**Affiliations:** ^1^Mental Health Unit, Finnish Institute for Health and Welfare, Helsinki, Finland; ^2^Department of Psychology, Northwestern University, Evanston, IL, United States; ^3^Department of Psychology, University of Maryland, Baltimore County, Baltimore, MD, United States; ^4^Department of Psychological Science, University of California, Irvine, Irvine, CA, United States

**Keywords:** psychotic experiences, perceptual abnormalities, PQ-B, differential item functioning (DIF), measurement invariance (MI), young adults, psychopathology

## Abstract

Valid measurement of group differences in self-reported psychotic-like experiences (PLEs) requires knowing any group-specific measurement properties of the instruments. We investigated the measurement invariance of the 21-item Prodromal Questionnaire–Brief (PQ-B) questionnaire across gender, ethnic minority/majority status, and presence of depressive symptoms in two different US non-clinical undergraduate samples (*N* = 1,099). For each item, endorsement of the experience and the associated distress were combined for analysis. A unidimensional model of the PQ-B fit the data well. Across genders, the PQ-B showed configural and metric, but not full scalar invariance; there were statistically significant differences in eight thresholds of six items, most being higher endorsement thresholds for self-identified females. Partial scalar invariance was also found for ethnic status, with five thresholds of three items being higher for the minority participants. For depressive symptomatology, defined as the top quintile by the Beck Depression Inventory–II, partial scalar invariance required dropping one item, after which there were statistically significant differences only in two response thresholds. Overall, a wide range of PLE questionnaire items were found to be robust to gender and ethnicity effects, strengthening confidence in found group differences in PLEs. Although full scalar invariance could not be ascertained for any of the group comparisons, the few found scalar differences across groups were small, with minimal impact on group PLE estimates. However, since PLEs are easily conceptually entangled with depression symptoms, similar items should be considered for exclusion if separable constructs are the target of investigation.

## Introduction

Psychotic-like experiences (PLEs) are abnormal perceptions or thoughts that resemble the positive symptoms of psychotic disorders. These include primarily hallucinations and illusions (for example, hearing voices, or tactile sensations such as that of bugs crawling on the skin) and delusions (such as paranoia, milder persecutory thoughts, or thoughts of reference). PLEs are present in ~ 8% of the general population (1), and people experiencing PLEs share etiological, demographic, and psychopathological risk factors with those with psychotic disorders (2). A richer understanding of PLEs can facilitate a more complete picture of the entire psychosis spectrum. Regardless of their relation with future psychosis, elucidating factors related to PLEs remains important as they have been associated with help-seeking behavior (3), impaired functioning (4–6), psychopathology and psychiatric diagnoses (7–9), as well as later hospitalization and suicidality (10–13).

Though semi-structured interviews are the gold standard for detecting clinically relevant PLEs thought to indicate psychosis risk, several questionnaires have been developed for first-stage clinical screening, which are in content very similar to questionnaires for measuring PLEs in the general population. Consequently, PLE questionnaires have been used for first-stage screening of psychosis risk even in the general population (14). Careful examination of the measurement biases in such instruments is therefore warranted.

Quantitative differences in PLEs across various groups have been found in both clinical and non-clinical settings. For example, studies suggest differences in the amount and severity of PLEs across ethnic groups, suggesting an association between ethnic minority status and PLEs (15–17). Gender differences have also been reported in PLEs, suggesting varying patterns of expression between men and women (7, 18–21). Moreover, PLEs have been found to be more prevalent among people suffering from non-psychotic mental health problems, such as depression or anxiety disorders (22, 23).

Although some of these group differences appear to be rather robust, the possibility remains that they are partly artifacts. This is because potential differences in measurement of PLE in various subpopulations are chiefly unknown. It is therefore possible that the observed differences in item scores and sum scores— or a lack thereof— could be attributed to differential functioning of the measure in the groups instead of the groups' actual features. For example, items assessing PLE endorsement and distress can be understood, interpreted, and answered differently depending on the respondent's characteristics, such as gender, health status, or cultural background. This has been found to be true with other common psychological measures, such as self-reports of depressive symptoms [for a review, see (24)], irrespective of the actual level of symptoms. For a questionnaire to be validly used to answer any substantive questions regarding group differences, its psychometric properties must be same across groups.

In order to ensure psychometric similarity of two measurements, it is necessary to assess both the structure and item-level characteristics of the measure, that is, both properties of the scale as a whole and properties of the individual items. This is not possible in the framework of classical test theory, which only makes use of indices of overall measure performance, such as sums of item scores, and does not consider that the same sum score can represent many different combinations of item scores. Latent trait models, in contrast, allow assessment of the measurement characteristics on the level of items and their unique properties, making it possible to consider and to compare the measurement model of the measure as well as the structure of the estimated latent construct (25).

Measurement invariance refers to the similar functioning of items across groups, in other words, to the similarity of item parameters of the measure in different groups or points of time. When groups interact in different ways with the items, differences may arise in the associations between the items and the latent construct (s). When these differences are sufficiently large, different constructs are actually being measured, making comparisons between the groups meaningless or even fallacious if the differing measurement properties are not accounted for.

Structural invariance, in contrast, refers to similarity of the structure of the latent construct across groups, i.e., the means, variance, and covariance between the dimensions of the latent trait of interest (25). Although these are often assessed by comparing the characteristics of the sum scores, structural similarity between different groups can be meaningfully assessed only if at least partial measurement invariance is confirmed first. Moreover, assessing the properties of latent traits instead of sums of item scores results in more accurate estimates of phenomena, especially at the extremes of the trait (26), which are often the clinically relevant ranges.

Thus, the assessment of measurement invariance is especially critical across groups that have been found to differ in the trait of interest. Among studies addressing PLEs questionnaires, measurement invariance has been demonstrated across gender (27, 28), an encouraging result which warrants replication. When extending the concept of PLEs to include schizotypy, results have been mixed with regards to ethnicity (29–32), and the non-invariance between ethnic groups may wholly or in part be explained by language version differences (31). Another critical distinction which has received little attention is the role of depressive symptomatology. As PLEs appear more frequently among those with depressive symptoms (23), and general-population psychopathology has even been conceptualized as a single factor (33), it is still unclear to what degree responses to PLE items are driven by general psychopathology rather than specific experiences. Measurement invariance between those with and without psychopathology has been suggested for one measure of PLE (34), but their findings leave room for interpretation, as they applied loose criteria.

Therefore, to validate self-reported PLE assessment, the current study primarily aims to assess the measurement and structural invariance of PLE across gender, ethnic minority status, and self-reported level of depression. The current study uses the Prodromal Questionnaire, Brief version (PQ-B), a short and well-established measure for detecting PLEs [for a review, see (35)]. Although the PQ-B has been shown to function well as a measure of PLEs (36–38), its psychometric features have not yet been thoroughly investigated, which is a secondary aim of the current study. Specifically, we hypothesized that the PQ-B is measurement invariant across gender, ethnic minority status and self-reported level of depression. Secondarily, we hypothesized that the PQ-B is essentially unidimensional and has adequate internal consistency. We employ a procedure in which nested models for compared groups are generated by adding model constraints, and successive models are assessed for worsening fit. As a reference analysis, the measurement invariance between two collection sites is also studied.

## Methods

### Participants

The self-report data (*N* = 1,103) were combined from two separate data sets from studies recruiting pre-degree psychology students. The participants of the first group (Site 1, *n* = 410) were recruited between November 2010 and May 2014 at the University of Colorado Boulder's Adolescent Development and Preventive Treatment (ADAPT) research program. Participants were young adults (aged 18 and older) in the University of Colorado Boulder's human subject recruitment pool (consisting of students and community members from the general population), and there were no exclusion criteria. The protocol and informed consent procedures were approved by the University Institutional Review Board.

The second group (Site 2, *n* = 693) consisted of undergraduate students recruited between November 2010 and May 2014 from introductory psychology courses at the University of Maryland, Baltimore County (UMBC). Participants were recruited as part of a larger study aimed at assessing undergraduate emotional, behavioral, and personality characteristics. Inclusion criteria noted that all participants must be over the age of 18. There were no additional exclusion criteria. All participants were offered extra credit for their participation, and the study was approved by the UMBC Institutional Review Board.

All subjects gave written informed consent in accordance with the Declaration of Helsinki (39).

### Measures

#### PQ-B

Psychotic-like symptoms were assessed using the PQ-B (40), which consists of 21 items. The PQ-B is an abbreviation and refinement of the Prodromal Questionnaire [PQ-92; (40)], with added distress assessment, as distressing symptoms are thought to be more relevant in psychosis spectrum measurement. The items reflect symptoms and experiences that can appear in those meeting criteria for psychosis risk syndromes. Appropriate cut-offs vary widely by use scenario and desired sensitivity (35); among help-seeking outpatients Xu et al. (41) suggested cut-offs of 7 and 24 for the total and distress scores, respectively, to ensure sufficient sensitivity and specificity. As in the present study, the measure has also been used to examine PLEs in the general population (42–44).

Each PQ-B item is first rated based on whether one has ever experienced a symptom (yes / no), and then according to how much distress the symptom causes, on a five-point scale (strongly disagree / disagree / neutral / agree / strongly agree; coded 1– 5). The symptoms sum score is the number of endorsed symptoms and the distress score is the mean of the coded distress ratings (in calculating these scores missing values were substituted with intra-individual means).

For the current study, all paired PQ-B symptom and distress item responses were transformed into single categorical variables, with a “No symptoms” response as the lowest level below the distress response alternatives. A preliminary nominal factor modeling indicated that “Strongly Disagree” and “Disagree” responses were not distinguishable on a unidimensional latent scale, and these were therefore collapsed. Due to infrequent responses, the categories (starting from “Strongly Agree”) were collapsed as necessary into the previous one, for each group comparison separately, to ensure a minimum of five responses for each alternative in each group; the number of categories per item were thus 3– 5 (mean 4.0 across all analyses). The collapsing and final number of categories for the items in each analysis is presented in Supplemental Table 1.

#### BDI-II

Self-reported depression was assessed with the second edition of the Beck Depression Inventory [BDI-II; (45)], which has 21 items scored 0–3. Items 16 and 18 were rescored to represent the amount of change in appetite and sleep, respectively, regardless of direction. Groups were defined as high (highest 20 %) vs. low depressive symptomatology (lowest 80 %) based on factor scores from a confirmatory factor analysis – group sizes were chosen beforehand to ensure sufficient statistical power for measurement invariance testing. For the purpose of reporting sum scores, missing values were substituted with intra-individual means.

### Sample Notes

For the minority status analyses, achieving adequate comparison group sizes required dichotomizing self-reported group membership to majority and minority, that is, Caucasian or not. The site comparisons included only Caucasian participants, to avoid confounding with minority status, as there was a large difference in the proportion of minority participants between the sites (17 vs. 66 % for Sites 1 and 2, respectively). Genders were used in the original self-reported format, as male or female only. We excluded the four respondents from Site 2 with all PQ-B responses missing, for a final *N* = 1,099 used in analyses. No outliers were removed from the data. Due to the nature of the undergraduate subject pool design, missing responses among the remaining respondents were rare across both sites (0.13 % of PQ-B endorsement responses and 1.1 % of distress ratings), with no individual having more than three missing endorsement responses.

### Statistical Methods

All analyses were conducted using Mplus v. 7.4 (46).

#### Confirmatory Factor Analysis

To assess the suitability of the assumption of unidimensionality, a single-dimensional item factor solution was computed using weighted least squares mean-adjusted (WLSM) estimation for categorical items (theta parametrization), which has been shown to be an accurate method of estimation (47). Missing values were treated as being at random, and were not substituted, as partial missingness of a respondent's data is allowed in this type of analysis. Model adequacy was quantified with the comparative fit index (CFI; values > 0.95 indicating good fit) and root mean square error of approximation (RMSEA; values < 0.05 indicating good fit) indices (48, 49); the weighted root mean square residual (WRMR; values < 1.0 indicating good fit) is shown as a descriptive measure (50). McDonald's ω is reported as an indicator of internal consistency, as recommended by Dunn et al. (51).

#### Procedure for Assessment of Measurement Invariance

Measurement invariance and structural invariance was assessed for site, gender, minority status, and self-reported level of depression. The procedure used, which was presented by Lesa Hoffman (personal communication, January 1st, 2017), is a modification of the procedure detailed by Millsap (25), differing from the original by not designating fixed anchor items. This procedure for categorical items is similar to an approach used in corresponding models with linear associations between the items and the latent trait (52). As in the confirmatory factor analysis, missing values were not substituted. Respondents' latent factor scores were estimated with *expected a posteriori* (EAP) scoring. High- and low-depression groups were formed in a balanced way by combining the top 20 % and low 80 % scoring individuals in each of the four gender-by-minority subgroups.

We tested configural, metric, scalar, and residual variance invariance, in this order. This was done by adding model constraints and by testing successive models for worsening fit. Nested models were compared using the DIFFTEST procedure, a modification to the χ^2^ test (53). Models were considered invariant if the increase in model misfit of the item factor model to the polychoric correlation matrix among the items was not significant at *p* < 0. 01. If the decrease in model fit was significant, parameters of individual items were freed to differ across groups in the order suggested by the modification indices, to achieve partial measurement invariance, if possible.

##### Configural Invariance

Initially, a unidimensional baseline model was estimated in each group simultaneously. The factor variance was fixed to unity (1) and the factor mean was fixed to zero in each group for identification, and then all item factor loadings (one per item) and thresholds (e.g., three per item given four response options) were estimated. The residual variances were fixed to 1 in both groups, as they cannot be uniquely identified in the configural invariance model. This approach presented applicable to ordered categorical responses by Lesa Hoffman (54) differs from the Millsap (25) procedure, which requires designating fixed “anchor” items.

##### Metric (Weak) Invariance

The metric invariance model enabled examining the equality of the unstandardized item factor loadings between groups. The factor variance was fixed to 1 in the first group for identification but was freely estimated in the other, and the factor mean was fixed to 0 in both groups for identification. Factor loadings were fixed to be equal across groups, item thresholds were estimated, and residual variances were fixed to 1. If necessary, item factor loadings were freed one at a time in order of decreasing modification index magnitude until partial metric invariance was confirmed.

##### Scalar (Strong) Invariance

Scalar invariance refers to the equality of the unstandardized item thresholds across groups, and this was examined by freeing the appropriate factor loadings across groups if partial metric invariance had been achieved. The factor mean and variance were fixed in one group for identification, but freed in the other. As for metric invariance, successive partial scalar invariance models were estimated, if necessary.

##### Residual Variance (Strict) Invariance

Finally, a residual variance invariance model was used to examine equality of the unstandardized residual variances across groups. The residual variances in the reference group were all fixed to 1 for identification, and the rest of the model parameters were estimated as described for the last (partial) scalar invariance model. A model with all residual variances freely estimated in the other group was fitted first, and then compared with a model in which all residual variances were fixed to 1.

## Results

### Sample Characteristics

Sample characteristics are presented in Table 1. The proportion of minority participants and the proportion of those with high depression scores was smaller at site 1. The sites were similar in gender, age, and PQ-B sum score distributions. Missingness was minimal both for demographic and questionnaire data. Mean reported PQ-B Distress was low, in that responses were tending toward “Disagree” rather than “Agree” to the follow-up item on distress associated with the symptom. Mean Distress was only moderately associated with the reported number of endorsed PQ-B symptoms (rank order correlation 0.37); as an illustration, the mean ± SD Distress was 2.3 ± 0.9 for the middle endorsement quintile (3 ≤ score ≤ 4.2) and 2.9 ± 0.7 for the top quintile (score > 8).

**Table 1:** Descriptives and missingness of observations on the study variables.

		**Descriptives**			**Missingness**		
		**Site 1**	**Site 2**	**Combined**	**Site 1**	**Site 2**	**Combined**
Participants	*n*	410	693	1,103	–	–	–
Minority ethnicity		17.4%	65.9%	47.8%	3.2 %	3.6 %	3.4 %
Female gender		58.2%	55.0%	56.2%	0.2 %	2.2 %	1.5 %
High Depression score		12.4%	24.5%	20.0%	0.0 %	0.4 %	0.3 %
Age (years)	M (SD)	19.5 (1.9)	20.0 (3.1)	19.8 (2.7)	0.5 %	1.7 %	1.3 %
BDI-II sum score^*^	M (SD)	7.0 (7.4)	10.1 (8.9)	9.0 (8.5)	0.0 %	0.4 %	0.4 %
PQ-B symptom sum score	M (SD)	4.4 (3.7)	4.7 (4.5)	4.6 (4.2)	0.0 %	0.6 %	0.4 %
PQ-B distress score mean	M (SD)	2.4 (0.9)	2.5 (0.9)	2.5 (0.9)	0.6 %	0.9 %	0.8 %

* *BDI-II sum scores estimated only for individuals with less than half of responses missing*.

### Unidimensional Model Fit

The confirmatory factor analysis of the PQ-B with combined endorsement and distress categories indicated good model fit for the unidimensional model, CFI = 0.953, RMSEA = 0.044 (90 % C.I.: 0.040–0.048), explained common variance 41.4 %. The model parameters are reported in Supplementary Table 3. The internal consistency of the scale was good, with McDonald's ω = 0.94.

### Measurement and Structural Invariance Across Collection Sites

In the site comparisons, the configural and metric invariance models were found to have good fit (results are presented in detail in Tables 2, 3). The metric model did not have significantly worse fit than the configural model, indicating metric invariance. The initial scalar invariance model (scalar model A) was found to have good fit, but significantly worse fit than the metric invariance model. To ameliorate the fit, the first threshold of item 16 was freed (scalar model B) as the modification indices suggested it to be the most substantial source of misfit. This resulted in good model fit, with non-significant increase in model misfit from the metric model, and partial scalar invariance was thus confirmed. Freeing all residuals resulted in better fit than the partial scalar invariance model with fixed residuals, and partial residual variance invariance was investigated next. As suggested by the modification indices, the residual variance was freed for item 13 (residuals fixed model B), which again resulted in good model fit but still showed a significant decrease in model fit. Freeing the residual variance also for item 12 (residual fixed model C) resulted in good fit along with non-significant decrease in model fit from the partial scalar invariance model, and partial residual variance invariance could be demonstrated.

**Table 2:** Measurement invariance by data collection site.

	**Model fit indicators**			**DIFFTEST**		
**Measurement invariance model**	**RMSEA (90% C.I.)**	**CFI**	**WRMR**	*****χ**^2^***	**df**	***p***
Configural	0.042 (0.038, 0.047)	0.958	1.54	–	–	–
Metric	0.038 (0.034, 0.043)	0.963	1.67	36.3	20	0.014
Scalar A	0.037 (0.032, 0.041)	0.961	1.76	94.2	62	0.005
Scalar, partial B	0.036 (0.032, 0.040)	0.963	1.74	78.1	61	0.069
Residual free	0.035 (0.031, 0.040)	0.965	1.64	–	–	–
Residual fixed A	0.036 (0.032, 0.040)	0.963	1.74	49.2	21	<0.001
Residual fixed, partial B	0.035 (0.031, 0.040)	0.964	1.73	44.9	20	0.001
Residual fixed, partial C	0.035 (0.031, 0.039)	0.965	1.71	34.9	19	0.015

**Table 3:** Parameters freed to achieve measurement invariance between data collection sites (in the order of freeing).

			**Cohen's**	**DIFFTEST**	**Modif**.
**Parameter freed**	**Site 1**	**Site 2**	***d***	***p***	index^**^
PQ16 Body changed; 1st threshold, No vs. (Strongly) Disagree	0.9	1.4	0.22	0.069	14.0
PQB13 Nonexistence; residual variance	1^*^	1.7	0.11	0.001	17.7
PQB12 Something wrong with mind; residual variance	1^*^	1.8	0.13	0.015	17.0

* *Fixed at unity*;

** *in previous model*.

In the assessment of structural invariance, significantly greater variability in the latent factor was found in participants assessed at site 2 (factor SD = 1.30) as compared to the participants of site 1 (factor SD fixed at 1). In the partial measurement invariance model, factor means did not significantly differ (Cohen's *d* = 0.08, *p* = 0.13).

### Measurement and Structural Invariance by Gender

In the gender comparisons, the configural as well as the metric invariance models were also found to have good fit (results are presented in detail in Tables 4, 5). As significant increase in model misfit was not found, metric invariance was confirmed. The scalar invariance model showed good fit, but the decrease in model misfit was significant, and therefore thresholds were freed one by one in the order of decreasing modification indices (Table 5). This resulted in successive scalar invariance models B–H. Finally, partial scalar invariance model H showed both good fit as well as non-significant increase in model misfit compared to the metric model, indicating partial scalar measurement invariance.

**Table 4:** Measurement invariance by gender.

**Measurement invariance model**	**Model fit indicators**			**DIFFTEST**		
	**RMSEA (90% C.I.)**	**CFI**	**WRMR**	*****χ**^2^***	**df**	***p***
Configural	0.042 (0.038, 0.047)	0.958	1.50	–	–	–
Metric	0.038 (0.033, 0.042)	0.964	1.62	33.5	20	0.030
Scalar A	0.038 (0.034, 0.043)	0.957	1.79	167.2	70	<0.001
Scalar, partial H	0.036 (0.031, 0.040)	0.963	1.71	88.4	63	0.019
Residual free	0.036 (0.031, 0.040)	0.965	1.60	–	–	–
Residual fixed A	0.036 (0.031, 0.040)	0.963	1.71	50.0	21	<0.001
Residual fixed B	0.035 (0.030, 0.039)	0.965	1.67	33.3	20	0.031

**Table 5:** Parameters freed to achieve measurement invariance between men and women (in the order of freeing).

**Parameter freed**	**Male**	**Female**	**Cohen's**	**DIFFTEST**	Modif. index^*^
			***d***	***p***	
PQB07 Special gifts, 1st threshold [No vs. (Strongly) Disagree]	0.8	1.3	0.28	<0.001	17.7
PQB06 Rambling speech, 1st threshold [No vs. (Strongly) Disagree]	0.7	0.3	0.24	<0.001	15.3
PQB15 Bizarre beliefs, 1st threshold [No vs. (Strongly) Disagree]	0.6	0.9	0.18	<0.001	7.5
PQB13 Nonexistence, 1st threshold [No vs. (Strongly) Disagree]	1.4	1.8	0.15	<0.001	5.7
PQB07 Special gifts, 2nd threshold [(Strongly) Disagree vs. Neutral]	1.6	2.0	0.17	0.002	5.7
PQB21 Speech sometimes hard to understand, 1st threshold [No vs. (Strongly) Disagree]	0.8	1.1	0.14	0.007	5.4
PQB13 Nonexistence, 3rd threshold (Neutral vs. Agree)	2.1	2.5	0.15	0.019	4.5
PQB14 Reality confusion, residual variance	1^*^	2.0	0.17	0.031	26.1

* *In previous model*.

Partial scalar invariance model with free residuals showed good fit and also significantly better fit than the model with residuals fixed. As suggested by modification indices, residual variance was freed for item 14 “Reality confusion” across groups, which resulted in good model fit and non-significant increase in model misfit, indicating partial residual variance measurement invariance.

Men and women were not found to differ in terms of variance of the latent factor (*p* = 0.01, factor SD for women was 1.16 when fixed to 1 for men), and the factor mean was slightly higher among women (Cohen's *d* = 0.01, *p* < 0.01).

### Measurement and Structural Invariance by Minority Status

**Figure 1:**
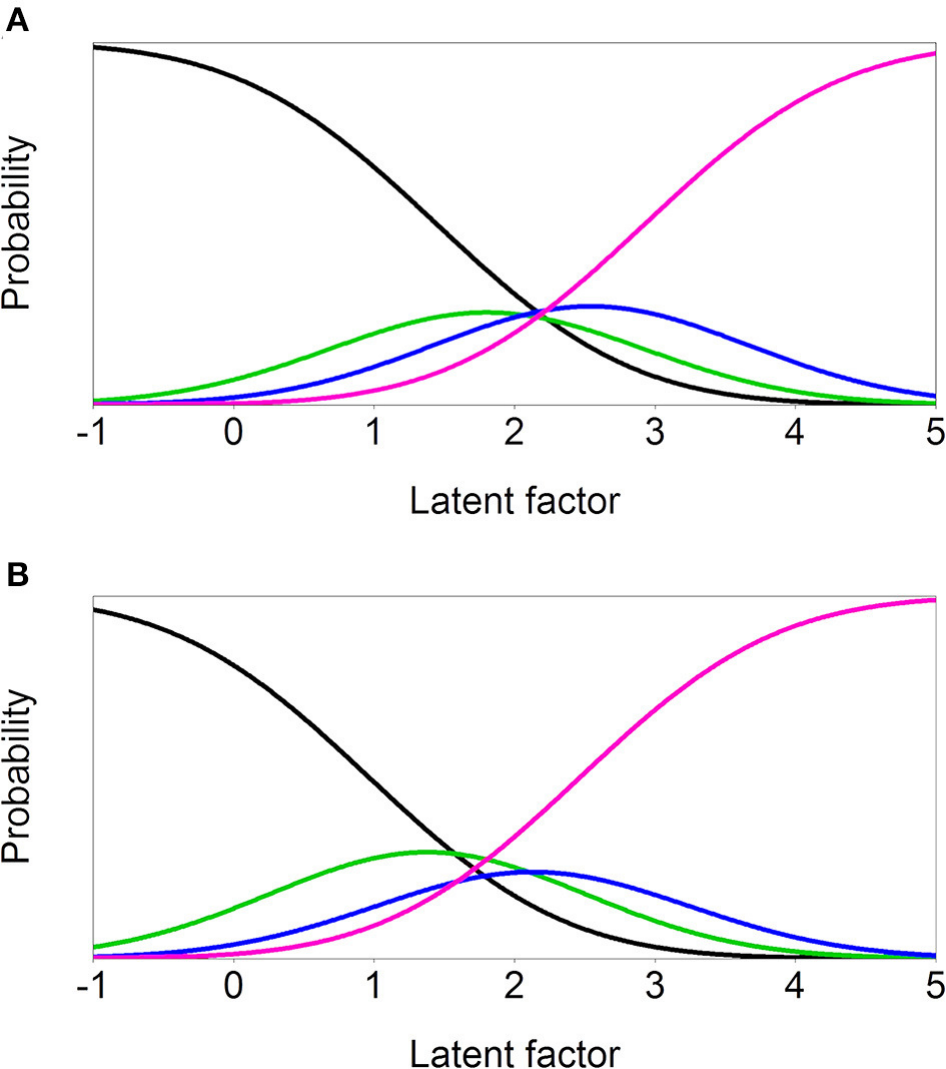
Item characteristic curves of the least invariant item 17 ‘Thoughts almost audible’ in the ethnic group MI comparison. **(A)** Minority **(B)** Majority.

Metric invariance by minority status was confirmed as the metric model did not show significantly worse model fit than the configural model (results are presented in detail in Tables 6, 7). After freeing all thresholds of item 17 “Thoughts almost audible,” together with the last threshold (“Agree” vs. “Strongly Agree”) of items 10 “Suddenly distracted” and 11 “Invisible force around,” partial scalar invariance was indicated by scalar model F having good fit, also relative to the metric model. Item characteristic curves for the least invariant item 17 “Thoughts almost audible” are presented in Figure 1. Residual variance invariance was confirmed without having to free any residual variances across groups, as initial residual fixed model was found to have good fit and the increase in model misfit was non-significant. As the groups did not differ in terms of factor variance or factor means, structural invariance could be confirmed.

**Table 6:** Measurement invariance by minority status.

Measurement invariance model	**Model fit indicators**			**DIFFTEST**		
	**RMSEA (90% C.I.)**	**CFI**	**WRMR**	*****χ**^2^***	**df**	***p***
Configural	0.040 (0.036, 0.045)	0.959	1.45	–	–	–
Metric	0.037 (0.032, 0.041)	0.964	1.59	35.8	20	0.016
Scalar A	0.036 (0.032, 0.041)	0.959	1.72	133.1	69	<0.001
Scalar, partial F	0.035 (0.030, 0.039)	0.963	1.68	90.0	64	0.018
Residual free	0.036 (0.031, 0.040)	0.963	1.60	–	–	–
Residual fixed	0.035 (0.030, 0.039)	0.963	1.68	35.7	21	0.023

**Table 7:** Parameters freed to achieve measurement invariance across minority status (in the order of freeing).

**Parameter freed**	**Majority**	**Minority**	**Cohen's**	**DIFFTEST**	Modif. index^*^
			***d***	***p***	
PQB17 Thoughts almost audible, 1st threshold [No vs. (Strongly) Disagree]	0.9	1.4	0.22	<0.001	12.4
PQB11 Invisible force around, 3rd threshold [Neutral vs. (Strongly) Agree]	1.6	2.0	0.21	0.001	10.3
PQB17 Thoughts almost audible, 2nd threshold [(Strongly) Disagree vs. Neutral]	2.2	2.7	0.15	0.004	5.4
PQB10 Suddenly distracted, 3rd threshold (Neutral vs. Agree)	2.1	2.5	0.15	0.008	5.0
PQB17 Thoughts almost audible, 3rd threshold (Neutral vs. Agree)	2.2	2.7	0.14	0.018	4.9

* *In previous model*.

### Measurement and Structural Invariance by Self-Reported Level of Depression

The BDI-II responses fit the unidimensional item factor model fairly well [CFI = 0.925, RMSEA = 0.074 (90% C.I. 0.070, 0.077), McDonald's ω = 0.89, Supplemental Table 4], and the factor score cut-off for determining high- and low-scoring groups corresponded approximately to a sum score of 16.1, in the middle of the “mild depression” range (the linear correlation between factor scores and sum scores was 0.95). Freeing residual covariance parameters between item pairs 4 & 12 and 15 & 20 (estimated residual covariances 0.27 and 0.23, respectively) improved model fit somewhat [CFI 0.942, RMSEA 0.065 (90% C.I. 0.061, 0.069)], and this model was used to calculate depression factor scores and assign group membership.

All depression group models were found to have good fit (Tables 8, 9). As only a non-significant increase in model misfit was detected for the metric invariance model, metric measurement invariance was confirmed. The initial scalar invariance model showed significantly worse fit as compared to the metric model, and freeing successive thresholds showed that item 12 “Something wrong with mind” was the major source of misfit: this item was therefore dropped from the depression subgroup analyses, and scalar invariance was investigated without it. Again, the initial model misfit required that thresholds were freed in successive partial scalar invariance models (scalar models B–G).

**Table 8:** Measurement invariance by high/low BDI factor scores.

**Measurement invariance model**	**Model fit indicators**			**DIFFTEST**		
	**RMSEA (90% C.I.)**	**CFI**	**WRMR**	*****χ**^2^***	**df**	***p***
Configural	0.041 (0.037, 0.046)	0.945	1.51	–	–	–
Metric	0.037 (0.032, 0.041)	0.954	1.63	31.5	20	0.049
Scalar A	0.038 (0.034, 0.042)	0.943	1.82	171.8	71	<0.001
Scalar G	0.035 (0.031, 0.040)	0.950	1.74	106.9	65	0.001

**Table 9:** Parameters freed to achieve measurement invariance by self-reported level of depression (in the order of freeing).

**Item, parameter freed**	**Low**	**High**	**Cohen's**	**DIFFTEST**	Modif. index^*^
			***d***	***p***	
PQB12 Something wrong with mind, 4th threshold (Agree vs. Strongly Agree)	3.4	2.7	0.13	<0.001	6.4
PQB12 Something wrong with mind, 2nd threshold [(Strongly) Disagree vs. Neutral]	1.5	0.9	0.24	<0.001	6.6
PQB12 Something wrong with mind, 3rd threshold (Neutral vs. Agree)	1.9	1.4	0.20	<0.001	5.8
PQB12 Something wrong with mind, 1st threshold [No vs. (Strongly) Disagree]	1.2	0.6	0.26	<0.001	5.8
PQB01 Familiar surroundings strange, 2nd threshold [(Strongly) Disagree vs. Neutral]	2.1	1.6	0.16	<0.001	5.5
PQB16 Body changed, 1st threshold [No vs. (Strongly) Disagree]	1.3	1.8	0.23	<0.001	4.0

* *In previous model*.

## Discussion

The aim of the current study was to assess the measurement and structural invariance of psychotic-like experiences (PLEs) across genders, ethnic minority status, and self-reported level of depression. As a reference analysis, the measurement invariance between two collection sites was also studied. PLEs were assessed using the PQ-B [for a review, see (35)]. The present psychometric results are to our knowledge the most detailed to date for this widely used instrument. The differences found in the measurement of PLEs across different groups were overall rather small, and the items had similar measurement properties across groups. However, some larger differences in the measurement of PLEs were detected, especially between the high and low depression groups.

In all comparisons, full metric invariance was achieved. In other words, the items were similarly related to the latent factor among men and women, in different ethnic groups, as well as among depressed and non-depressed participants, and also across participating sites. However, in all comparisons, minor differences were found in the thresholds of responses, meaning that the same response category was indicative of somewhat different levels of latent phenomena/PLEs across groups. Note that there was only one detected threshold difference across sites, where group differences were hypothesized to be negligible. Considering the large number of statistical comparisons, this single finding could be attributed to chance.

### Ethnicity

Item 17 “Thoughts almost audible” was measured differently among the ethnic majority (white Caucasians) as compared to the group of ethnic minority participants, as all thresholds of this item had to be allowed to differ across groups for partial invariance. If using only item 17 “Thoughts almost audible,” psychotic-like symptom severity would be underestimated for the minority. However, the differences of threshold position on the standardized latent factor scale are small (< 0.5 SD), despite being statistically significant in this fairly large sample. This variable should therefore be dropped when ethnicity is of focal interest, but the effect is likely diluted to practical insignificance when using the entire PQ-B for other purposes. Also, some of the thresholds of items 10 “Suddenly distracted” and 11 “Invisible force around” had to be freed across groups, as the ethnic majority participants were more inclined to report these symptoms as distressing. Unfortunately, sample size considerations precluded analyses by minority subgroup.

Previous studies have found PLEs questionnaires to show greater non-invariance across ethnic groups, but our results— which used a single questionnaire version— support the notion that those findings may be partially attributed to differences between language versions. In contrast, one study using a single version of the PQ-B (55), reported strong invariance across ethnic groups, but their respondents were aged 9–10, which suggests that the minor ethnic group non-invariance found in the present study emerges in adolescence or adulthood.

### Gender

Some differences were found in the measurement of PLEs among men and women. The threshold between responses “No” vs. “[Strongly] disagree” had to be allowed to differ for a total of 5 items (items 7 “Special gifts”; 6 “Rambling speech”; 15 “Bizarre beliefs”; 13 “Nonexistence”; 21 “Speech sometimes hard to understand”). Women were more inclined to report “Rambling speech” and reported experiencing it at a lower latent trait level. Conversely, men reported more readily “Special gifts,” “Bizarre beliefs,” “Nonexistence” and “Speech sometimes hard to understand.” In addition, gender differences were found in reporting of distress caused by symptoms of the item 7 “Special gifts” and 13 “Nonexistence,” as men were more inclined to report more distress caused by these symptoms.

These results differed from a previous study by Fonseca-Pedrero et al. (28), where PQ-B item endorsement showed strong measurement invariance across gender among community adolescents. Nevertheless, they simultaneously report large gender effects for items 7 and 20 in a separate differential item functioning (DIF) analysis, and we thus replicated their results for item 7, despite a different age group and language version. In another study using the Youth Psychosis At-Risk Questionnaire – Brief (YPARQ-B) among community adolescents, Fonseca-Pedrero et al. (27) also reported full measurement invariance across gender. However, of the five PQ-B items for which we found endorsement non-invariance, only the content of items 13 and 15 is assessed by the YPARQ-B, and furthermore, the effects in the present study were small (Cohen's *d* 0.15 and 0.18, respectively). The discrepancy in results is potentially attributable to PLE items being less clinically relevant among younger age groups.

### Depression

Item 12 “Something wrong with mind” was found to be answered differently in high and low depression groups. Reporting to have experienced this symptom indicated a lower amount of PLEs in the high depression group, which likely reflects the unspecific mental health connotations of the item. There was also a difference in reporting item 16 “Body changed” between high- and low depression groups, which is understandable, as body dysmorphia is one of the symptoms of depression and also included in the BDI. Since items with this kind of content partially depend on depression levels, they should be considered for exclusion when the intention is to measure PLEs specifically, rather than, for example, screening for psychosis risk, where depression is a relevant risk factor of its own, and conceptual disentanglement is less critical.

Our results did not conflict with the findings of Siddi et al. (34), as the Launay–Slade Hallucinations Scale-Extended version does not include a non-specific item corresponding to PQ-B item 12, or an item that can be interpreted as body dysmorphia.

### Study Strengths and Limitations

The age distribution of the participants is appropriate for the assessment of psychotic-like experiences. Also, as the data was a combination of two different datasets from two research groups, and the results can be assumed to be more robust than results obtained from one single dataset. The comparison across sites also allowed an illustration of the upper boundary of chance findings.

Some further issues should be considered when interpreting the results. First, both sites were in the US, and the results may not be directly generalized to other countries. On the other hand, this allowed a language- and culture-sharing comparison between ethnic groups. Second, the responses are limited to the scale of the measure used. Though the PQ-B scale is more informative than dichotomous responses, it may be that, for instance, a concretely anchored frequency scale would allow more precise measurement, with larger effect sizes for group differences. Another aspect of the PQ-B, as used in a general-population sample, is that most of its variance is in a non-clinical range, and measurement invariance in the clinically relevant extreme with distress may be masked. The PQ-B is, however, representative of existing PLE questionnaires, and effect sizes were all quite small. Third, as the participants were all university students, the findings are not necessarily directly generalizable to a broader general population. However, we have no specific reason to assume that the approach adopted in the study would be sensitive to education effects. Fourth, there were relevant demographic characteristics of our sample we did not ascertain, preventing us from fully characterizing our convenience sample. Future studies extending this work would be well served to consider some of these factors to facilitate generalization beyond college samples (e.g., socio-economic status, previous and current psychological treatment, university major area of study, etc.). Fifth, it is possible that the highly sensitive and personal content of the items might have resulted in a bias toward socially desirable responding and a tendency to under-report PLE. Nevertheless, as the data was collected completely anonymously, and also because of the analytical approach adopted, this is not likely to cause substantial bias in the findings.

Finally, we did not validate the PQ-B against interviews or similar questionnaires, but the content validity of the PQ questionnaires has been previously established (35), even in children (55).

### Conclusions and Clinical Implications

In sum, a wide range of PLE questionnaire items were found to be robust to gender and ethnicity effects, strengthening confidence in found group differences. In contrast, since PLEs are easily conceptually entangled with depression symptoms, similar items should be considered for exclusion if separable constructs are the target of investigation.

The findings, which suggested important conceptual and methodological considerations when formulating and interpreting PLE findings in the general population, also suggest that researchers and clinicians should be mindful of measurement invariance when employing PLE questionnaires to the assessment of clinical populations, such as those at clinical high-risk for psychosis. Though we were only able to test the influence of one other symptom dimension, depression, our results show that responding to some PLE items is clearly skewed by non-PLE symptomatology. Before clinical application, our study would need to be replicated in a relevant clinical sample. Future work would also be well served to conduct similar investigations on similar and commonly adopted measures.

## Data Availability Statement

The data analyzed in this study is subject to the following licenses/restrictions: anonymized data available on request, without undue reservation. Requests to access these datasets should be directed to Vijay Mittal, vijay.mittal@northwestern.edu, or Jason Schiffman, schiffma@umbc.edu, respectively.

## Ethics Statement

The studies involving human participants were reviewed and approved by the University of Colorado Boulder Institutional Review Board and the UMBC Institutional Review Board. The participants provided their written informed consent to participate in this study.

## Author Contributions

UL and ST were responsible for the data analysis and drafting of the manuscript. VM and JS provided the previously collected data. All authors contributed to project design, interpretation of the findings, and revising the manuscript.

## Conflict of Interest

The authors declare that the research was conducted in the absence of any commercial or financial relationships that could be construed as a potential conflict of interest.
