# Supplementary material for: Measurement Invariance of Psychotic-Like Symptoms as Measured With the Prodromal Questionnaire, Brief Version (PQ-B) in Adolescent and Adult Population Samples
Source: Front Psychiatry. 2021 Jan 21;11:593355. doi: 10.3389/fpsyt.2020.593355 (PMC7873944; doi:10.3389/fpsyt.2020.593355)
Supplement: Supplementary file 1 [file Data_Sheet_1.PDF]

Supplementary materials

Contents

Supplemental tables 2
Response frequencies 2
Recoding of responses 3
PQ-B item-factor model 8
BDI-II item-factor model 9
Mplus scripts for Ethnicity MI analyses 10
Shared part 10
Model-specific statements 10
Configural model 10
Metric model 11
Scalar model 13
Partial Scalar model B 15
Partial Scalar model C 17
Partial Scalar model D 19
Partial Scalar model E 21
Partial Scalar model F 23
Residual invariance, baseline model 25
Residual invariance, model with residuals fixed 27
Structural invariance, variances 29
Structural invariance, means 31

Supplemental tables

Response frequencies

Supplemental Table 1. PQ-B Item endorsement and distress rating percentages.

	Endorsement			Distress ratings					
PQ-B item	Yes *n*	Yes %	Missing *n*	Strongly Disagree	Disagree	Neutral	Agree	Strongly Agree	Missing
1	151	13.7 %	0	9.3 %	21.9 %	33.8 %	31.8 %	3.3 %	0.0 %
2	338	30.8 %	0	24.6 %	28.4 %	25.4 %	17.5 %	3.0 %	1.2 %
3	117	10.6 %	3	18.8 %	30.8 %	31.6 %	16.2 %	0.0 %	2.6 %
4	117	10.6 %	1	41.0 %	28.2 %	19.7 %	9.4 %	1.7 %	0.0 %
5	171	15.6 %	0	11.7 %	14.6 %	26.3 %	36.8 %	9.4 %	1.2 %
6	404	36.8 %	2	25.0 %	22.8 %	23.5 %	22.5 %	4.7 %	1.5 %
7	210	19.1 %	1	48.1 %	17.1 %	20.5 %	8.6 %	3.3 %	2.4 %
8	386	35.1 %	1	15.0 %	17.6 %	28.0 %	31.6 %	7.5 %	0.3 %
9	196	17.8 %	1	9.2 %	21.4 %	30.1 %	32.7 %	5.6 %	1.0 %
10	291	26.5 %	0	20.3 %	25.8 %	32.3 %	18.6 %	2.1 %	1.0 %
11	273	24.8 %	3	16.5 %	23.4 %	23.1 %	30.4 %	5.5 %	1.1 %
12	297	27.0 %	0	6.1 %	13.8 %	26.6 %	41.8 %	11.1 %	0.7 %
13	127	11.6 %	2	11.0 %	20.5 %	33.9 %	22.0 %	11.0 %	1.6 %
14	334	30.4 %	1	14.4 %	18.3 %	32.3 %	26.3 %	6.6 %	2.1 %
15	308	28.0 %	1	33.4 %	26.6 %	27.6 %	8.8 %	2.9 %	0.6 %
16	189	17.2 %	2	20.1 %	21.2 %	28.0 %	26.5 %	4.2 %	0.0 %
17	242	22.0 %	0	25.2 %	28.1 %	27.3 %	14.9 %	3.3 %	1.2 %
18	398	36.2 %	1	10.8 %	23.6 %	26.9 %	30.7 %	6.5 %	1.5 %
19	91	8.3 %	2	18.7 %	28.6 %	25.3 %	19.8 %	5.5 %	2.2 %
20	84	7.6 %	0	26.2 %	25.0 %	27.4 %	16.7 %	3.6 %	1.2 %
21	302	27.5 %	1	21.2 %	20.2 %	29.1 %	20.5 %	6.3 %	2.6 %

Recoding of responses

Supplemental Tables 2 a-e. Recoding of responses to achieve sufficient number of observations in each category for the various analyses.

a) unidimensional model

Item	No	Strongly Disagree	Disagree	Neutral	Agree	Strongly Agree
1	0	1	1	2	3	4
2	0	1	1	2	3	4
3	0	1	1	2	3	3
4	0	1	1	2	3	4
5	0	1	1	2	3	4
6	0	1	1	2	3	4
7	0	1	1	2	3	4
8	0	1	1	2	3	4
9	0	1	1	2	3	4
10	0	1	1	2	3	4
11	0	1	1	2	3	4
12	0	1	1	2	3	4
13	0	1	1	2	3	4
14	0	1	1	2	3	4
15	0	1	1	2	3	4
16	0	1	1	2	3	4
17	0	1	1	2	3	4
18	0	1	1	2	3	4
19	0	1	1	2	3	4
20	0	1	1	2	3	4
21	0	1	1	2	3	4

b) comparison across measurement sites

Item	No	Strongly Disagree	Disagree	Neutral	Agree	Strongly Agree
1	0	1	1	2	3	3
2	0	1	1	2	3	3
3	0	1	1	2	2	2
4	0	1	1	2	2	2
5	0	1	1	2	3	3
6	0	1	1	2	3	3
7	0	1	1	2	2	2
8	0	1	1	2	3	4
9	0	1	1	2	3	3
10	0	1	1	2	3	3
11	0	1	1	2	3	4
12	0	1	1	2	3	4
13	0	1	1	2	3	3
14	0	1	1	2	3	3
15	0	1	1	2	3	3
16	0	1	1	2	3	3
17	0	1	1	2	3	3
18	0	1	1	2	3	4
19	0	1	1	2	3	3
20	0	1	1	2	2	2
21	0	1	1	2	3	3

c) comparison across genders

Item	No	Strongly Disagree	Disagree	Neutral	Agree	Strongly Agree
1	0	1	1	2	2	2
2	0	1	1	2	2	2
3	0	1	1	2	2	2
4	0	1	1	2	2	2
5	0	1	1	2	2	2
6	0	1	1	2	3	3
7	0	1	1	2	2	2
8	0	1	1	2	3	3
9	0	1	1	2	2	2
10	0	1	1	2	2	2
11	0	1	1	2	3	3
12	0	1	1	2	3	3
13	0	1	1	2	3	3
14	0	1	1	2	3	3
15	0	1	1	2	2	2
16	0	1	1	2	2	2
17	0	1	1	2	2	2
18	0	1	1	2	3	3
19	0	1	1	2	2	2
20	0	1	1	2	2	2
21	0	1	1	2	3	3

d) comparison across ethnicity status

Item	No	Strongly Disagree	Disagree	Neutral	Agree	Strongly Agree
1	0	1	1	2	3	3
2	0	1	1	2	3	3
3	0	1	1	2	3	4
4	0	1	1	2	2	2
5	0	1	1	2	3	4
6	0	1	1	2	3	4
7	0	1	1	2	3	3
8	0	1	1	2	3	4
9	0	1	1	2	3	3
10	0	1	1	2	3	3
11	0	1	1	2	3	3
12	0	1	1	2	3	4
13	0	1	1	2	3	4
14	0	1	1	2	3	4
15	0	1	1	2	3	3
16	0	1	1	2	3	3
17	0	1	1	2	3	3
18	0	1	1	2	3	4
19	0	1	1	2	3	3
20	0	1	1	2	3	3
21	0	1	1	2	3	4

e) comparison across high/low depression groups

Item	No	Strongly Disagree	Disagree	Neutral	Agree	Strongly Agree
1	0	1	1	2	3	3
2	0	1	1	2	3	3
3	0	1	1	2	3	3
4	0	1	1	2	2	2
5	0	1	1	2	3	4
6	0	1	1	2	3	4
7	0	1	1	2	3	3
8	0	1	1	2	3	4
9	0	1	1	2	3	4
10	0	1	1	2	3	3
11	0	1	1	2	3	4
12	0	1	1	2	3	4
13	0	1	1	2	3	4
14	0	1	1	2	3	4
15	0	1	1	2	3	3
16	0	1	1	2	3	3
17	0	1	1	2	3	3
18	0	1	1	2	3	4
19	0	1	1	2	3	3
20	0	1	1	2	3	3
21	0	1	1	2	3	4

PQ-B item-factor model

Supplemental Table 3. Standardized one-dimensional item-factor model of combined endorsement/distress PQ-B responses.

		Thresholds			
Item	Loading	1<br>No<br>–<br>Strongly<br>Disagree	2<br>Strongly<br>Disagree<br>–<br>Disagree	3<br>Disagree<br>–<br>Neutral	4<br>Neutral<br>–<br>(Strongly)<br>Agree
1	0.68	1.09	1.31	1.66	2.61
2	0.57	0.51	1.07	1.53	2.36
3	0.63	1.26	1.63	2.11	*
4	0.49	1.25	1.84	2.26	2.91
5	0.65	1.02	1.21	1.46	2.18
6	0.69	0.35	0.89	1.28	2.11
7	0.52	0.89	1.54	2.00	2.49
8	0.69	0.38	0.72	1.09	1.94
9	0.57	0.93	1.16	1.49	2.33
10	0.68	0.64	1.08	1.60	2.54
11	0.61	0.68	1.05	1.34	2.21
12	0.73	0.62	0.79	1.07	1.88
13	0.63	1.21	1.42	1.77	2.23
14	0.64	0.53	0.84	1.28	2.05
15	0.62	0.59	1.22	1.84	2.40
16	0.60	0.95	1.28	1.62	2.44
17	0.67	0.78	1.28	1.75	2.44
18	0.68	0.36	0.73	1.10	1.98
19	0.65	1.40	1.73	2.03	2.61
20	0.72	1.44	1.79	2.16	2.78
21	0.73	0.61	1.02	1.45	2.11

* Value not available, as this item’s two highest remaining categories were collapsed.

BDI-II item-factor model

Supplemental Table 4. Standardized one-dimensional item-factor model of BDI-II responses.

Item	Loading	Thresholds 0–1	1–2	2–3
1	0.76	0.45	1.91	2.40
2	0.71	0.19	1.54	2.18
3	0.69	0.18	1.11	2.21
4	0.70	0.51	1.64	2.40
5	0.64	0.31	1.59	2.26
6	0.62	0.95	1.63	2.14
7	0.81	0.44	1.11	1.78
8	0.69	0.00	0.99	1.75
9	0.69	1.04	2.49	2.91
10	0.62	0.75	1.52	1.81
11	0.66	0.30	1.64	2.13
12	0.72	0.45	1.60	2.16
13	0.64	0.34	1.22	1.58
14	0.84	0.88	1.37	2.23
15	0.77	0.07	1.50	2.21
16	0.48	-0.46	0.92	2.11
17	0.70	0.57	1.66	2.40
18	0.54	-0.02	1.26	1.97
19	0.66	0.14	1.19	2.21
20	0.74	-0.10	1.39	2.11
21	0.41	1.17	1.85	2.44

Fit information for the BDI-II model:
CFI 0.925, RMSEA 0.074 (90% C.I. 0.070, 0.077), explained common variance 46 %.
Freeing residual covariance parameters between item pairs 4 & 12 and 15 & 20 (estimated residual covariances 0.27 and 0.23, respectively) improved model fit to CFI 0.942, RMSEA 0.065 (90% C.I. 0.061, 0.069).

Mplus scripts for Ethnicity MI analyses

Shared part

```
DATA:
! Data sets are slightly different for each group comparison
! due to collapsing of categories
  FILE IS "PQ-B_Ethnicity.dat";
VARIABLE:
  NAMES ARE ID age gender ethnicity PQB01-PQB21;
  USEVARIABLES ARE PQB01-PQB21;
  CATEGORICAL ARE PQB01-PQB21;
  IDVARIABLE IS ID;
  MISSING ARE .;
  GROUPING IS ethnicity (1 = majority 0 = minority);
ANALYSIS:
  ESTIMATOR IS WLSMV;
  PARAMETERIZATION=THETA;
```

Model-specific statements

Configural model

```
! REFERENCE GROUP CONFIGURAL MODEL

MODEL:
! Factor loadings all estimated
PQB_f BY PQB01-PQB21*;
! Item intercepts (all free)
[PQB01$1-PQB21$1*];
[PQB01$2-PQB21$2*];
[PQB01$3-PQB03$3*]; ! 4 categories in all items except for item 4
[PQB05$3-PQB21$3*]; ! 4 categories in all items except for item 4
[PQB05$4*]; ! 5 categories only in these
[PQB06$4*]; ! 5 categories only in these
[PQB08$4*]; ! 5 categories only in these
[PQB12$4*]; ! 5 categories only in these
[PQB13$4*]; ! 5 categories only in these
[PQB14$4*]; ! 5 categories only in these
[PQB18$4*]; ! 5 categories only in these
[PQB21$4*]; ! 5 categories only in these
! Residual variances (all fixed)
PQB01-PQB21@1;
! Factor mean and variance fixed in categorical configural model for
identification
[PQB_f@0]; PQB_f@1;

! CONFIGURAL MODEL FOR SECOND GROUP

MODEL minority:
! Factor loadings all estimated
PQB_f BY PQB01-PQB21*;
```

Metric model

```
! REFERENCE GROUP METRIC MODEL;

MODEL:
! Factor loadings all estimated but same across groups
PQB_f BY
PQB01* (L01)
PQB02* (L02)
PQB03* (L03)
PQB04* (L04)
PQB05* (L05)
PQB06* (L06)
PQB07* (L07)
PQB08* (L08)
PQB09* (L09)
PQB10* (L10)
PQB11* (L11)
PQB12* (L12)
PQB13* (L13)
PQB14* (L14)
PQB15* (L15)
PQB16* (L16)
PQB17* (L17)
PQB18* (L18)
PQB19* (L19)
PQB20* (L20)
PQB21* (L21)
;
! Item intercepts (all free)
[PQB01$1-PQB21$1*];
[PQB01$2-PQB21$2*];
[PQB01$3-PQB03$3*]; ! 4 categories in all items except for item 4
[PQB05$3-PQB21$3*]; ! 4 categories in all items except for item 4
[PQB05$4*]; ! 5 categories only in these
[PQB06$4*]; ! 5 categories only in these
[PQB08$4*]; ! 5 categories only in these
[PQB12$4*]; ! 5 categories only in these
[PQB13$4*]; ! 5 categories only in these
[PQB14$4*]; ! 5 categories only in these
[PQB18$4*]; ! 5 categories only in these
[PQB21$4*]; ! 5 categories only in these
! Residual variances (all fixed)
PQB01-PQB21@1;
! Factor mean and variance fixed for identification
[PQB_f@0]; PQB_f@1;
```

```
! METRIC MODEL FOR SECOND GROUP

MODEL minority:
! Factor loadings all estimated but same across groups
PQB_f BY
PQB01* (L01)
PQB02* (L02)
PQB03* (L03)
PQB04* (L04)
PQB05* (L05)
PQB06* (L06)
PQB07* (L07)
PQB08* (L08)
PQB09* (L09)
PQB10* (L10)
PQB11* (L11)
PQB12* (L12)
PQB13* (L13)
PQB14* (L14)
PQB15* (L15)
PQB16* (L16)
PQB17* (L17)
PQB18* (L18)
PQB19* (L19)
PQB20* (L20)
PQB21* (L21)
;
! Item intercepts (all free)
[PQB01$1-PQB21$1*];
[PQB01$2-PQB21$2*];
[PQB01$3-PQB03$3*]; ! 4 categories in all items except for item 4
[PQB05$3-PQB21$3*]; ! 4 categories in all items except for item 4
[PQB05$4*]; ! 5 categories only in these
[PQB06$4*]; ! 5 categories only in these
[PQB08$4*]; ! 5 categories only in these
[PQB12$4*]; ! 5 categories only in these
[PQB13$4*]; ! 5 categories only in these
[PQB14$4*]; ! 5 categories only in these
[PQB18$4*]; ! 5 categories only in these
[PQB21$4*]; ! 5 categories only in these
! Residual variances (all fixed)
PQB01-PQB21@1;
! Factor mean STILL FIXED and variance NOW FREE
[PQB_f@0]; PQB_f*;


OUTPUT:  STDYX CINTERVAL SVALUES;

SAVEDATA:  RESULTS IS PQ_ethn_metric_results.txt;
           DIFFTEST=MetricA.dat; ! Save metric info

PLOT:  TYPE IS PLOT3;
```

Scalar model

```
! REFERENCE GROUP SCALAR MODEL;

MODEL:
! Factor loadings all estimated but same across groups
PQB_f BY
PQB01* (L01)
PQB02* (L02)
PQB03* (L03)
PQB04* (L04)
PQB05* (L05)
PQB06* (L06)
PQB07* (L07)
PQB08* (L08)
PQB09* (L09)
PQB10* (L10)
PQB11* (L11)
PQB12* (L12)
PQB13* (L13)
PQB14* (L14)
PQB15* (L15)
PQB16* (L16)
PQB17* (L17)
PQB18* (L18)
PQB19* (L19)
PQB20* (L20)
PQB21* (L21)
;
! Item intercepts (all free but equal)
[PQB01$1-PQB21$1*];
[PQB01$2-PQB21$2*];
[PQB01$3-PQB03$3*]; ! 4 categories in all items except for item 4
[PQB05$3-PQB21$3*]; ! 4 categories in all items except for item 4
[PQB05$4*]; ! 5 categories only in these
[PQB06$4*]; ! 5 categories only in these
[PQB08$4*]; ! 5 categories only in these
[PQB12$4*]; ! 5 categories only in these
[PQB13$4*]; ! 5 categories only in these
[PQB14$4*]; ! 5 categories only in these
[PQB18$4*]; ! 5 categories only in these
[PQB21$4*]; ! 5 categories only in these
! Residual variances fixed for identification
PQB01-PQB21@1;
! Factor mean and variance fixed in categorical configural model for
identification
[PQB_f@0]; PQB_f@1;
```

```
! SCALAR MODEL FOR SECOND GROUP

MODEL minority:
! Factor loadings all estimated but same across groups
PQB_f BY
PQB01* (L01)
PQB02* (L02)
PQB03* (L03)
PQB04* (L04)
PQB05* (L05)
PQB06* (L06)
PQB07* (L07)
PQB08* (L08)
PQB09* (L09)
PQB10* (L10)
PQB11* (L11)
PQB12* (L12)
PQB13* (L13)
PQB14* (L14)
PQB15* (L15)
PQB16* (L16)
PQB17* (L17)
PQB18* (L18)
PQB19* (L19)
PQB20* (L20)
PQB21* (L21)
;
! Item intercepts held EQUAL if unspecified
! Residual variances fixed for identification
PQB01-PQB21@1;
! Factor mean NOW FREE and variance STILL FREE
PQB_f*; [PQB_f*];


OUTPUT:  STDYX CINTERVAL SVALUES;

SAVEDATA:  RESULTS IS PQ_ethn__scalar_results.txt;
           DIFFTEST=ScalarA.dat; ! Save scalar info

PLOT:  TYPE IS PLOT3;
```

Partial Scalar model B

```
! REFERENCE GROUP SCALAR MODEL;

MODEL:
! Factor loadings all estimated but same across groups
PQB_f BY
PQB01* (L01)
PQB02* (L02)
PQB03* (L03)
PQB04* (L04)
PQB05* (L05)
PQB06* (L06)
PQB07* (L07)
PQB08* (L08)
PQB09* (L09)
PQB10* (L10)
PQB11* (L11)
PQB12* (L12)
PQB13* (L13)
PQB14* (L14)
PQB15* (L15)
PQB16* (L16)
PQB17* (L17)
PQB18* (L18)
PQB19* (L19)
PQB20* (L20)
PQB21* (L21)
;
! Item intercepts (all free but equal)
[PQB01$1-PQB21$1*];
[PQB01$2-PQB21$2*];
[PQB01$3-PQB03$3*]; ! 4 categories in all items except for item 4
[PQB05$3-PQB21$3*]; ! 4 categories in all items except for item 4
[PQB05$4*]; ! 5 categories only in these
[PQB06$4*]; ! 5 categories only in these
[PQB08$4*]; ! 5 categories only in these
[PQB12$4*]; ! 5 categories only in these
[PQB13$4*]; ! 5 categories only in these
[PQB14$4*]; ! 5 categories only in these
[PQB18$4*]; ! 5 categories only in these
[PQB21$4*]; ! 5 categories only in these
! Residual variances fixed for identification
PQB01-PQB21@1;
! Factor mean and variance fixed in categorical configural model for
identification
[PQB_f@0]; PQB_f@1;
```

```
! SCALAR MODEL FOR SECOND GROUP

MODEL minority:
! Factor loadings all estimated but same across groups
PQB_f BY
PQB01* (L01)
PQB02* (L02)
PQB03* (L03)
PQB04* (L04)
PQB05* (L05)
PQB06* (L06)
PQB07* (L07)
PQB08* (L08)
PQB09* (L09)
PQB10* (L10)
PQB11* (L11)
PQB12* (L12)
PQB13* (L13)
PQB14* (L14)
PQB15* (L15)
PQB16* (L16)
PQB17* (L17)
PQB18* (L18)
PQB19* (L19)
PQB20* (L20)
PQB21* (L21)
;
! Item intercepts held EQUAL if unspecified
! One threshold freed
[PQB17$1*];
! Residual variances fixed for identification
PQB01-PQB21@1;
! Factor mean NOW FREE and variance STILL FREE
PQB_f*; [PQB_f*];


OUTPUT:  STDYX CINTERVAL SVALUES;

SAVEDATA:  RESULTS IS PQ_ethn_ScalarB_results.txt;
           DIFFTEST=ScalarB.dat; ! Save scalar info

PLOT:  TYPE IS PLOT3;
```

Partial Scalar model C

```
! REFERENCE GROUP SCALAR MODEL;

MODEL:
! Factor loadings all estimated but same across groups
PQB_f BY
PQB01* (L01)
PQB02* (L02)
PQB03* (L03)
PQB04* (L04)
PQB05* (L05)
PQB06* (L06)
PQB07* (L07)
PQB08* (L08)
PQB09* (L09)
PQB10* (L10)
PQB11* (L11)
PQB12* (L12)
PQB13* (L13)
PQB14* (L14)
PQB15* (L15)
PQB16* (L16)
PQB17* (L17)
PQB18* (L18)
PQB19* (L19)
PQB20* (L20)
PQB21* (L21)
;
! Item intercepts (all free but equal)
[PQB01$1-PQB21$1*];
[PQB01$2-PQB21$2*];
[PQB01$3-PQB03$3*]; ! 4 categories in all items except for item 4
[PQB05$3-PQB21$3*]; ! 4 categories in all items except for item 4
[PQB05$4*]; ! 5 categories only in these
[PQB06$4*]; ! 5 categories only in these
[PQB08$4*]; ! 5 categories only in these
[PQB12$4*]; ! 5 categories only in these
[PQB13$4*]; ! 5 categories only in these
[PQB14$4*]; ! 5 categories only in these
[PQB18$4*]; ! 5 categories only in these
[PQB21$4*]; ! 5 categories only in these
! Residual variances fixed for identification
PQB01-PQB21@1;
! Factor mean and variance fixed in categorical configural model for
identification
[PQB_f@0]; PQB_f@1;
```

```
! SCALAR MODEL FOR SECOND GROUP

MODEL minority:
! Factor loadings all estimated but same across groups
PQB_f BY
PQB01* (L01)
PQB02* (L02)
PQB03* (L03)
PQB04* (L04)
PQB05* (L05)
PQB06* (L06)
PQB07* (L07)
PQB08* (L08)
PQB09* (L09)
PQB10* (L10)
PQB11* (L11)
PQB12* (L12)
PQB13* (L13)
PQB14* (L14)
PQB15* (L15)
PQB16* (L16)
PQB17* (L17)
PQB18* (L18)
PQB19* (L19)
PQB20* (L20)
PQB21* (L21)
;
! Item intercepts held EQUAL if unspecified
! Three thresholds freed
[PQB17$1*];
[PQB11$3*];
! Residual variances fixed for identification
PQB01-PQB21@1;
! Factor mean NOW FREE and variance STILL FREE
PQB_f*; [PQB_f*];


OUTPUT:  STDYX CINTERVAL SVALUES;

SAVEDATA:  RESULTS IS PQ_ethn_ScalarC_results.txt;
           DIFFTEST=ScalarC.dat; ! Save scalar info

PLOT:  TYPE IS PLOT3;
```

Partial Scalar model D

```
! REFERENCE GROUP SCALAR MODEL;

MODEL:
! Factor loadings all estimated but same across groups
PQB_f BY
PQB01* (L01)
PQB02* (L02)
PQB03* (L03)
PQB04* (L04)
PQB05* (L05)
PQB06* (L06)
PQB07* (L07)
PQB08* (L08)
PQB09* (L09)
PQB10* (L10)
PQB11* (L11)
PQB12* (L12)
PQB13* (L13)
PQB14* (L14)
PQB15* (L15)
PQB16* (L16)
PQB17* (L17)
PQB18* (L18)
PQB19* (L19)
PQB20* (L20)
PQB21* (L21)
;
! Item intercepts (all free but equal)
[PQB01$1-PQB21$1*];
[PQB01$2-PQB21$2*];
[PQB01$3-PQB03$3*]; ! 4 categories in all items except for item 4
[PQB05$3-PQB21$3*]; ! 4 categories in all items except for item 4
[PQB05$4*]; ! 5 categories only in these
[PQB06$4*]; ! 5 categories only in these
[PQB08$4*]; ! 5 categories only in these
[PQB12$4*]; ! 5 categories only in these
[PQB13$4*]; ! 5 categories only in these
[PQB14$4*]; ! 5 categories only in these
[PQB18$4*]; ! 5 categories only in these
[PQB21$4*]; ! 5 categories only in these
! Residual variances fixed for identification
PQB01-PQB21@1;
! Factor mean and variance fixed in categorical configural model for
identification
[PQB_f@0]; PQB_f@1;
```

```
! SCALAR MODEL FOR SECOND GROUP

MODEL minority:
! Factor loadings all estimated but same across groups
PQB_f BY
PQB01* (L01)
PQB02* (L02)
PQB03* (L03)
PQB04* (L04)
PQB05* (L05)
PQB06* (L06)
PQB07* (L07)
PQB08* (L08)
PQB09* (L09)
PQB10* (L10)
PQB11* (L11)
PQB12* (L12)
PQB13* (L13)
PQB14* (L14)
PQB15* (L15)
PQB16* (L16)
PQB17* (L17)
PQB18* (L18)
PQB19* (L19)
PQB20* (L20)
PQB21* (L21)
;
! Item intercepts held EQUAL if unspecified
! Four thresholds freed
[PQB17$1*];
[PQB11$3*];
[PQB17$2*];
! Residual variances fixed for identification
PQB01-PQB21@1;
! Factor mean NOW FREE and variance STILL FREE
PQB_f*; [PQB_f*];


OUTPUT:  STDYX CINTERVAL SVALUES;

SAVEDATA:  RESULTS IS PQ_ethn_ScalarD_results.txt;
           DIFFTEST=ScalarD.dat; ! Save scalar info

PLOT:  TYPE IS PLOT3;
```

Partial Scalar model E

```
! REFERENCE GROUP SCALAR MODEL;

MODEL:
! Factor loadings all estimated but same across groups
PQB_f BY
PQB01* (L01)
PQB02* (L02)
PQB03* (L03)
PQB04* (L04)
PQB05* (L05)
PQB06* (L06)
PQB07* (L07)
PQB08* (L08)
PQB09* (L09)
PQB10* (L10)
PQB11* (L11)
PQB12* (L12)
PQB13* (L13)
PQB14* (L14)
PQB15* (L15)
PQB16* (L16)
PQB17* (L17)
PQB18* (L18)
PQB19* (L19)
PQB20* (L20)
PQB21* (L21)
;
! Item intercepts (all free but equal)
[PQB01$1-PQB21$1*];
[PQB01$2-PQB21$2*];
[PQB01$3-PQB03$3*]; ! 4 categories in all items except for item 4
[PQB05$3-PQB21$3*]; ! 4 categories in all items except for item 4
[PQB05$4*]; ! 5 categories only in these
[PQB06$4*]; ! 5 categories only in these
[PQB08$4*]; ! 5 categories only in these
[PQB12$4*]; ! 5 categories only in these
[PQB13$4*]; ! 5 categories only in these
[PQB14$4*]; ! 5 categories only in these
[PQB18$4*]; ! 5 categories only in these
[PQB21$4*]; ! 5 categories only in these
! Residual variances fixed for identification
PQB01-PQB21@1;
! Factor mean and variance fixed in categorical configural model for
identification
[PQB_f@0]; PQB_f@1;
```

```
! SCALAR MODEL FOR SECOND GROUP

MODEL minority:
! Factor loadings all estimated but same across groups
PQB_f BY
PQB01* (L01)
PQB02* (L02)
PQB03* (L03)
PQB04* (L04)
PQB05* (L05)
PQB06* (L06)
PQB07* (L07)
PQB08* (L08)
PQB09* (L09)
PQB10* (L10)
PQB11* (L11)
PQB12* (L12)
PQB13* (L13)
PQB14* (L14)
PQB15* (L15)
PQB16* (L16)
PQB17* (L17)
PQB18* (L18)
PQB19* (L19)
PQB20* (L20)
PQB21* (L21)
;
! Item intercepts held EQUAL if unspecified
! Five thresholds freed
[PQB17$1*];
[PQB11$3*];
[PQB17$2*];
[PQB10$3*];
! Residual variances fixed for identification
PQB01-PQB21@1;
! Factor mean NOW FREE and variance STILL FREE
PQB_f*; [PQB_f*];


OUTPUT:  STDYX CINTERVAL SVALUES;

SAVEDATA:  RESULTS IS PQ_ethn_ScalarE_results.txt;
           DIFFTEST=ScalarE.dat; ! Save scalar info

PLOT:  TYPE IS PLOT3;
```

<u>Partial Scalar model F</u>

```
! REFERENCE GROUP SCALAR MODEL;

MODEL:
! Factor loadings all estimated but same across groups
PQB_f BY
PQB01* (L01)
PQB02* (L02)
PQB03* (L03)
PQB04* (L04)
PQB05* (L05)
PQB06* (L06)
PQB07* (L07)
PQB08* (L08)
PQB09* (L09)
PQB10* (L10)
PQB11* (L11)
PQB12* (L12)
PQB13* (L13)
PQB14* (L14)
PQB15* (L15)
PQB16* (L16)
PQB17* (L17)
PQB18* (L18)
PQB19* (L19)
PQB20* (L20)
PQB21* (L21)
;
! Item intercepts (all free but equal)
[PQB01$1-PQB21$1*];
[PQB01$2-PQB21$2*];
[PQB01$3-PQB03$3*]; ! 4 categories in all items except for item 4
[PQB05$3-PQB21$3*]; ! 4 categories in all items except for item 4
[PQB05$4*]; ! 5 categories only in these
[PQB06$4*]; ! 5 categories only in these
[PQB08$4*]; ! 5 categories only in these
[PQB12$4*]; ! 5 categories only in these
[PQB13$4*]; ! 5 categories only in these
[PQB14$4*]; ! 5 categories only in these
[PQB18$4*]; ! 5 categories only in these
[PQB21$4*]; ! 5 categories only in these
! Residual variances fixed for identification
PQB01-PQB21@1;
! Factor mean and variance fixed in categorical configural model for
identification
[PQB_f@0]; PQB_f@1;
```

```
! SCALAR MODEL FOR SECOND GROUP

MODEL minority:
! Factor loadings all estimated but same across groups
PQB_f BY
PQB01* (L01)
PQB02* (L02)
PQB03* (L03)
PQB04* (L04)
PQB05* (L05)
PQB06* (L06)
PQB07* (L07)
PQB08* (L08)
PQB09* (L09)
PQB10* (L10)
PQB11* (L11)
PQB12* (L12)
PQB13* (L13)
PQB14* (L14)
PQB15* (L15)
PQB16* (L16)
PQB17* (L17)
PQB18* (L18)
PQB19* (L19)
PQB20* (L20)
PQB21* (L21)
;
! Item intercepts held EQUAL if unspecified
! Five thresholds freed
[PQB17$1*];
[PQB11$3*];
[PQB17$2*];
[PQB10$3*];
[PQB17$3*];
! Residual variances fixed for identification
PQB01-PQB21@1;
! Factor mean NOW FREE and variance STILL FREE
PQB_f*; [PQB_f*];


OUTPUT:  STDYX CINTERVAL SVALUES;

SAVEDATA:  RESULTS IS PQ_ethn_ScalarF_results.txt;
           DIFFTEST=ScalarF.dat; ! Save scalar info

PLOT:  TYPE IS PLOT3;
```

Residual invariance, baseline model

```
! REFERENCE GROUP MODEL;

MODEL:
! Factor loadings all estimated but same across groups
PQB_f BY
PQB01* (L01)
PQB02* (L02)
PQB03* (L03)
PQB04* (L04)
PQB05* (L05)
PQB06* (L06)
PQB07* (L07)
PQB08* (L08)
PQB09* (L09)
PQB10* (L10)
PQB11* (L11)
PQB12* (L12)
PQB13* (L13)
PQB14* (L14)
PQB15* (L15)
PQB16* (L16)
PQB17* (L17)
PQB18* (L18)
PQB19* (L19)
PQB20* (L20)
PQB21* (L21)
;
! Item intercepts (all free but equal)
[PQB01$1-PQB21$1*];
[PQB01$2-PQB21$2*];
[PQB01$3-PQB03$3*]; ! 4 categories in all items except for item 4
[PQB05$3-PQB21$3*]; ! 4 categories in all items except for item 4
[PQB05$4*]; ! 5 categories only in these
[PQB06$4*]; ! 5 categories only in these
[PQB08$4*]; ! 5 categories only in these
[PQB12$4*]; ! 5 categories only in these
[PQB13$4*]; ! 5 categories only in these
[PQB14$4*]; ! 5 categories only in these
[PQB18$4*]; ! 5 categories only in these
[PQB21$4*]; ! 5 categories only in these
! Residual variances FIXED in this group
PQB01-PQB21@1;
! Factor mean and variance fixed for identification
[PQB_f@0]; PQB_f@1;
```

```
! MODEL FOR SECOND GROUP

MODEL minority:
! Factor loadings all estimated but same across groups
PQB_f BY
PQB01* (L01)
PQB02* (L02)
PQB03* (L03)
PQB04* (L04)
PQB05* (L05)
PQB06* (L06)
PQB07* (L07)
PQB08* (L08)
PQB09* (L09)
PQB10* (L10)
PQB11* (L11)
PQB12* (L12)
PQB13* (L13)
PQB14* (L14)
PQB15* (L15)
PQB16* (L16)
PQB17* (L17)
PQB18* (L18)
PQB19* (L19)
PQB20* (L20)
PQB21* (L21)
;
! Item intercepts (all EQUAL IF UNSPECIFIED)
[PQB17$1*];
[PQB11$3*];
[PQB17$2*];
[PQB10$3*];
[PQB17$3*];
! Residual variances FREE in this group
PQB01-PQB21*;
! Factor mean and variance free
PQB_f*; [PQB_f*];


OUTPUT:  STDYX CINTERVAL SVALUES;

SAVEDATA:  RESULTS IS PQ_ethn_residual_free_results.txt;
           DIFFTEST=Residual_free.dat; ! Save residual info

PLOT:  TYPE IS PLOT3;
```

Residual invariance, model with residuals fixed

```
! REFERENCE GROUP MODEL;

MODEL:
! Factor loadings all estimated but same across groups
PQB_f BY
PQB01* (L01)
PQB02* (L02)
PQB03* (L03)
PQB04* (L04)
PQB05* (L05)
PQB06* (L06)
PQB07* (L07)
PQB08* (L08)
PQB09* (L09)
PQB10* (L10)
PQB11* (L11)
PQB12* (L12)
PQB13* (L13)
PQB14* (L14)
PQB15* (L15)
PQB16* (L16)
PQB17* (L17)
PQB18* (L18)
PQB19* (L19)
PQB20* (L20)
PQB21* (L21)
;
! Item intercepts (all free but equal)
[PQB01$1-PQB21$1*];
[PQB01$2-PQB21$2*];
[PQB01$3-PQB03$3*]; ! 4 categories in all items except for item 4
[PQB05$3-PQB21$3*]; ! 4 categories in all items except for item 4
[PQB05$4*]; ! 5 categories only in these
[PQB06$4*]; ! 5 categories only in these
[PQB08$4*]; ! 5 categories only in these
[PQB12$4*]; ! 5 categories only in these
[PQB13$4*]; ! 5 categories only in these
[PQB14$4*]; ! 5 categories only in these
[PQB18$4*]; ! 5 categories only in these
[PQB21$4*]; ! 5 categories only in these
! Residual variances FIXED in this group
PQB01-PQB21@1;
! Factor mean and variance fixed for identification
[PQB_f@0]; PQB_f@1;
```

```
!  MODEL FOR SECOND GROUP

MODEL minority:
! Factor loadings all estimated but same across groups
PQB_f BY
PQB01* (L01)
PQB02* (L02)
PQB03* (L03)
PQB04* (L04)
PQB05* (L05)
PQB06* (L06)
PQB07* (L07)
PQB08* (L08)
PQB09* (L09)
PQB10* (L10)
PQB11* (L11)
PQB12* (L12)
PQB13* (L13)
PQB14* (L14)
PQB15* (L15)
PQB16* (L16)
PQB17* (L17)
PQB18* (L18)
PQB19* (L19)
PQB20* (L20)
PQB21* (L21)
;
! Item intercepts (all EQUAL IF UNSPECIFIED)
[PQB17$1*];
[PQB11$3*];
[PQB17$2*];
[PQB10$3*];
[PQB17$3*];
! Residual variances NOW FIXED in this group too
PQB01-PQB21@1;
! Factor mean and variance free
PQB_f*; [PQB_f*];


OUTPUT:  STDYX CINTERVAL SVALUES;

SAVEDATA:  RESULTS IS PQ_ethn_residual_fixed_results.txt;
           DIFFTEST=Residual_fixed.dat; ! Save residual info

PLOT:  TYPE IS PLOT3;
```

Structural invariance, variances

```
! REFERENCE GROUP MODEL;

MODEL:
! Factor loadings all equal
PQB_f BY
PQB01* (L01)
PQB02* (L02)
PQB03* (L03)
PQB04* (L04)
PQB05* (L05)
PQB06* (L06)
PQB07* (L07)
PQB08* (L08)
PQB09* (L09)
PQB10* (L10)
PQB11* (L11)
PQB12* (L12)
PQB13* (L13)
PQB14* (L14)
PQB15* (L15)
PQB16* (L16)
PQB17* (L17)
PQB18* (L18)
PQB19* (L19)
PQB20* (L20)
PQB21* (L21)
;
! Item intercepts (all free)
[PQB01$1-PQB21$1*];
[PQB01$2-PQB21$2*];
[PQB01$3-PQB03$3*]; ! 4 categories in all items except for item 4
[PQB05$3-PQB21$3*]; ! 4 categories in all items except for item 4
[PQB05$4*]; ! 5 categories only in these
[PQB06$4*]; ! 5 categories only in these
[PQB08$4*]; ! 5 categories only in these
[PQB12$4*]; ! 5 categories only in these
[PQB13$4*]; ! 5 categories only in these
[PQB14$4*]; ! 5 categories only in these
[PQB18$4*]; ! 5 categories only in these
[PQB21$4*]; ! 5 categories only in these
! Residual variances fixed for identification (in this group)
PQB01-PQB21@1;
! Factor mean and variance fixed for identification
PQB_f@1;[PQB_f@0];
```

```
! SCALAR MODEL FOR SECOND GROUP

MODEL minority:
! Factor loadings all equal
PQB_f BY
PQB01* (L01)
PQB02* (L02)
PQB03* (L03)
PQB04* (L04)
PQB05* (L05)
PQB06* (L06)
PQB07* (L07)
PQB08* (L08)
PQB09* (L09)
PQB10* (L10)
PQB11* (L11)
PQB12* (L12)
PQB13* (L13)
PQB14* (L14)
PQB15* (L15)
PQB16* (L16)
PQB17* (L17)
PQB18* (L18)
PQB19* (L19)
PQB20* (L20)
PQB21* (L21)
;
! Item intercepts held equal if unspecified
[PQB17$1*];
[PQB11$3*];
[PQB17$2*];
[PQB10$3*];
[PQB17$3*];
! Residual variances Fixed
PQB01-PQB21@1;
! Factor mean still free, variance NOW FIXED
PQB_f@1; [PQB_f*];


OUTPUT:  STDYX CINTERVAL SVALUES;

SAVEDATA:  RESULTS IS PQ_ethn_structural_variances_results.txt;
           DIFFTEST = Structural_variances.dat;

PLOT:  TYPE IS PLOT3;
```

Structural invariance, means

```
! REFERENCE GROUP MODEL;
MODEL:
! Factor loadings all equal
PQB_f BY
PQB01* (L01)
PQB02* (L02)
PQB03* (L03)
PQB04* (L04)
PQB05* (L05)
PQB06* (L06)
PQB07* (L07)
PQB08* (L08)
PQB09* (L09)
PQB10* (L10)
PQB11* (L11)
PQB12* (L12)
PQB13* (L13)
PQB14* (L14)
PQB15* (L15)
PQB16* (L16)
PQB17* (L17)
PQB18* (L18)
PQB19* (L19)
PQB20* (L20)
PQB21* (L21)
;
! Item intercepts (all free)
[PQB01$1-PQB21$1*];
[PQB01$2-PQB21$2*];
[PQB01$3-PQB03$3*]; ! 4 categories in all items except for item 4
[PQB05$3-PQB21$3*]; ! 4 categories in all items except for item 4
[PQB05$4*]; ! 5 categories only in these
[PQB06$4*]; ! 5 categories only in these
[PQB08$4*]; ! 5 categories only in these
[PQB12$4*]; ! 5 categories only in these
[PQB13$4*]; ! 5 categories only in these
[PQB14$4*]; ! 5 categories only in these
[PQB18$4*]; ! 5 categories only in these
[PQB21$4*]; ! 5 categories only in these
! Residual variances fixed for identification (in this group)
PQB01-PQB21@1;
! Factor mean and variance fixed for identification
[PQB_f@0]; PQB_f@1;
```

```
! MODEL FOR SECOND GROUP

MODEL minority:
! Factor loadings all equal
PQB_f BY
PQB01* (L01)
PQB02* (L02)
PQB03* (L03)
PQB04* (L04)
PQB05* (L05)
PQB06* (L06)
PQB07* (L07)
PQB08* (L08)
PQB09* (L09)
PQB10* (L10)
PQB11* (L11)
PQB12* (L12)
PQB13* (L13)
PQB14* (L14)
PQB15* (L15)
PQB16* (L16)
PQB17* (L17)
PQB18* (L18)
PQB19* (L19)
PQB20* (L20)
PQB21* (L21)
;
! Item intercepts held equal if unspecified
  [PQB17$1*];
  [PQB11$3*];
  [PQB17$2*];
  [PQB10$3*];
  [PQB17$3*];
! Residual variances FIXED
 PQB01-PQB21@1;
! Factor mean NOW FIXED, variance STILL fixed
PQB_f@1; [PQB_f@0];


OUTPUT:  STDYX CINTERVAL SVALUES;

SAVEDATA:  RESULTS IS PQ_ethn_structural_means_results.txt;

PLOT:  TYPE IS PLOT3;
```
